# The impact of gendered organizational systems on women’s career advancement

**DOI:** 10.3389/fpsyg.2015.00905

**Published:** 2015-06-30

**Authors:** Deborah A. O’Neil, Margaret M. Hopkins

**Affiliations:** ^1^Department of Management, College of Business Administration, Bowling Green State University, Bowling Green, OH, USA; ^2^Department of Management, College of Business and Innovation, University of Toledo, Toledo, OH, USA

**Keywords:** women leaders, gendered organizations, women’s career advancement, gender bias, women’s career development, women and confidence

## Abstract

In this Perspective article we propose that in order to pave the way for women’s career advancement into the senior ranks of organizations, attention must be directed at the systemic norms and structures that drive the gendered nature of the workplace. A focus on individual level issues, i.e., women lacking confidence and women opting out, detracts from the work that must be done at the organizational level in order to dismantle the system of pervasive, structural disadvantage facing women seeking to advance to senior leadership positions.

In the spring of 2014, two accomplished female journalists, Kay and Shipman (2014b), wrote an article in the *Atlantic Magazine* called *The Confidence Gap*. In this article they proposed that women’s lack of progress and inability to break through the glass ceiling in large numbers is due to what they termed “women’s acute lack of confidence.” They acknowledged that maternal instincts and cultural and institutional barriers may also be contributing factors, but stated that at the most basic level, confidence is the key missing ingredient to women’s success. They went on to publish a book on the subject, *The Confidence Code: The Science and Art of Self-Assurance—What Women Should Know*.

We believe that while it is true that some women may lack confidence, and indeed some men as well, painting all women with the broad brush of lacking in confidence ignores the societal, cultural and organizational norms that elevate men as natural leaders and regard women as “less than.” In this Perspective article we propose that the “women lack confidence” viewpoint relies on a “fix the women” perspective (c.f., Kottke and Agars, 2005; Wittenberg-Cox, 2013) rather than directing attention to the systemic norms and structures that drive the gendered nature of the workplace (Acker, 1991; Meyerson and Fletcher, 2000; Williams, 2000), described as the “male, competitive model” (Hewlett, 2007, p. 13). This reified model continues to regulate how men and women behave in organizational life. What may be perceived as women’s lack of confidence is in reality a pervasive, systemic disadvantage that women face in the work environment that serves to undermine them as they seek to advance into leadership positions. Suggesting that women don’t advance to the highest ranks of leadership because they lack confidence ignores the prevailing structures and systems (Vinnicombe et al., 2014) that exacerbate work/life balance concerns, and life/career stage issues (O’Neil et al., 2015) and thus continue to put women at a disadvantage. The cumulative effect of this positioning is that gendered organizational contexts remain firmly in place, and women remain under-represented at the highest ranks of our organizations.

Another characteristic of the gendered organizational system that negatively affects women in the workplace is second generation gender bias (Eagly and Carli, 2007; Ely et al., 2011; Ibarra et al., 2013), so named as it has mostly replaced overt discrimination with more subtle, less visible forms of prejudice. Cortina (2008) proposed that selective workplace incivility toward women and minorities is a form of this covert discrimination that has taken the place of overt sexism and racism. She assembled three theoretical streams of thought as to why this has occurred: First, these less visible forms of bias have replaced overt discrimination which is now seen as less desirable and in many cases illegal. Second, there is a long history of prejudice against women and minorities that has likely become more implicit than explicit over time, given changing historical and societal norms. Third, perhaps these more subtle forms of bias have existed all along, but were not as visible given that the more blatant forms of prejudice and discrimination overshadowed them. Unfortunately, the cumulative effect of these “incivilities” on women’s advancement into senior leadership positions is real and lasting.

Hoobler et al. (2011) group the various theoretical explanations for women’s lack of progress into four main areas: the effects of the barriers inherent in the glass ceiling, the time needed for enough women to progress through the pipeline, an evolutionary psychology perspective that suggests that women are not naturally suited for leadership, and finally that the nature of the “24/7 economy” is incompatible for women caring for families. These authors also provide an additional perspective related to the opportunities that women are or are not provided by managers in their organizations. They suggest that a vicious cycle of managers’ assessments of female workers as lower in career motivation results in lower career development opportunities being offered which leads to fewer women in senior level positions (Hoobler et al., 2014). “Women’s lack of ascension to higher management is at least partly explained by women not getting the opportunities and encouragement, that is, the critical organizational development, necessary to aspire to upper management positions” (p. 723).

As a result of these systemic factors, women’s lagging advancement into the ranks of senior leadership has been ascribed not only to a lack of confidence, but also to a personal choice to “opt out” (Belkin, 2003) or “off ramp” from their professional lives (Hewlett, 2007). These arguments focus on women self-selecting out of the work world due to personal choices involving family and care-giving and to viewing the costs of ascending to senior leadership roles as too high to pay in terms of the impact on their personal lives. The fact is that women may *choose* different paths because the traditional organizational route to the top does not support women simultaneously being accomplished careerists and responsible care-givers. To call these actions a matter of choice ignores the cumulative impact of decades spent slogging through challenging organizational contexts. In other words, this is a false choice. Slaughter (2012) wrote a piece in the *Atlantic Magazine*, entitled, *Why Women Still Can’t Have it All*. This article added fuel to the long simmering debate about whether women could successfully combine career and family. In this article she called out the standard of the male norm as detrimental to women and to organizations:

“If women are ever to achieve real equality as leaders, then we have to stop accepting male behavior and male choices as the default and the ideal. We must insist on changing social policies and bending career tracks to accommodate *our* choices, too.”

“Ultimately, it is society that must change, coming to value choices to put family ahead of work just as much as those to put work ahead of family. If we really valued those choices, we would value the people who make them; if we valued the people who make them, we would do everything possible to hire and retain them; if we did everything possible to allow them to combine work and family equally over time, then the choices would get a lot easier.”

Previous findings have shown that women have a tendency to hold themselves hyper-accountable for their successes and failures, absolving organizational systems for any part they may play in stalled careers (O’Neil et al., 2011). Women have also reported that they believe that they need to work harder and faster just to keep pace with men (Ragins et al., 1998). Add to these findings men’s documented tendencies toward over-confidence (Kay and Shipman, 2014a) and you have a perfect storm of conditions that conspire to paint a picture of women as lacking in confidence, opting out, and just generally not being cut out for demanding leadership positions. As Meyerson and Fletcher (2000) aptly noted, women have been blaming themselves for years for not fitting in, which further obfuscates the real root of the problem—that our organizational systems do not work for half of the working population. Wittenberg-Cox (2013) suggested that rather than rely on the glass ceiling metaphor, it is more apt to call what keeps women from leadership positions “gender asbestos,” which is making half the population disappear…” She called for the need to adapt “entire systems that were designed for one half of the human race to the reality, lives, and talents of the other half” (p. 110). Clearly the traditional male model of organizing is only increasing the gender gap in leadership roles.

In the ongoing debate about what keeps women from advancing into the highest levels of leadership, we believe that the focus needs to be on the organizational and societal levels not the individual level. As Meyerson and Fletcher (2000, p. 135) noted, “the problems are systemic, not individual.” Women developing all the confidence in the world, refusing to exit non-supportive organizational systems, and not blaming themselves for their lack of advancement won’t change the fact that the organizational deck is stacked against them combining productive work and family lives simultaneously. If we are to create the 21st century workforce with all the talent necessary to deal with the challenges of our global society, structures and systems must accommodate women’s lives, not the other way around.

Leadership can be found at all levels of the organizational hierarchy. However, senior leaders have the power to change organizational systems and structures. Thus, it is imperative to include female perspectives at the senior level in order to advocate for, inform, and educate all members of the organization, and to engender structural changes that foster a more inclusive environment for all workers. Also, women in senior leadership positions serve as role models and signal to women further down in the organizational hierarchy that organizational career paths can be open to them as well.

In their review of the literature on women’s careers O’Neil et al. (2008) proposed three reasons why organizational structures and systems are still firmly entrenched in the traditional, masculine form of organizing. First, they suggested that the current structures work for those employed at the senior levels of organizations, the majority of whom are men. Thus there is no compelling rationale for changing the system since the status quo works for those in charge. Second, they proposed that comprehensive data about women’s experiences at lower and middle levels of organizational life are not systematically collected. They posited that this means that women who may be poised to move up in the organizational hierarchy may simultaneously be juggling multiple life roles and finding organizational policies and procedures non-supportive of their life choices. This lack of organizational support, encouragement, and opportunities may bear some direct responsibility for women “opting out.” Thus, rather than suggest that there is something inherent in women that makes them exit organizational life, examining the impact of traditional masculine forms of organizing may be more effective in solving the problem of the lack of women in leadership roles. Third, they proposed that while structures and systems may change, organizational culture and individual attitudes often lag far behind. They highlighted Virginia Schein’s (1976; 2007) classic, “think manager, think male” studies that showed that from the 1970’s to the 2000’s, men’s attitudes about women being less suitable than men for leadership roles have remained firmly entrenched. These attitudes continue to put women aspiring to senior leadership roles at a distinct disadvantage, as evidenced by Burke’s et al (2008, p. 279) conclusion that “the biggest obstacle to career advancement for women is the attitudes, biases, perceptions and behaviors of their male colleagues.” See (Figure 1) for the impact of gendered organizations on women and why the status quo remains firmly entrenched.

**FIGURE 1:**
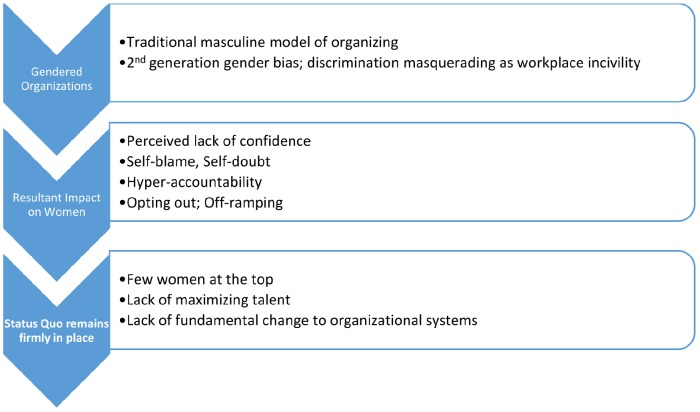
**The impact of gendered organizational systems on women’s career advancement**.

So what is the solution to this problem to which we seem to understand the contributing factors but have been thus far unable to solve effectively in large measure? We believe that we must keep the focus on breaking down and rebuilding the systems and structures that continue to keep women from ascending to the highest levels of our organizations. To do that we must have the courage to recognize, surface, and name the subtle acts of bias and discrimination that undermine women at all levels of our organizations. We must continue to question the organizational norms that have been in place since mid-last century when economic realities, social norms, and women’s roles were vastly different than they are today. We must stop blaming women for not fitting into a system that is rigged against them. Instead of suggesting that women should be more confident, more assertive, more out-spoken, more “fill in the blank,” let’s embrace women for who they are and the unique and valuable contributions they bring to organizational life. Let’s stop trying to fix the women, and fix the system instead. Finally, we must enlist all leaders, women and men, to understand the importance of developing the broadest, most diverse talent pool possible, and to finding workable solutions to make that happen. Perhaps only then can we hope to find more women advancing to senior leadership levels and engendering change in organizational policies and practices that will pave the way for more equitable, just organizational systems that work for both women and men.

## Conflict of Interest Statement

The authors declare that the research was conducted in the absence of any commercial or financial relationships that could be construed as a potential conflict of interest.
